# Mitigating creativity risks of knowledge hiding through GenAI knowledge seeking: moderating roles of goal orientation

**DOI:** 10.3389/fpsyg.2026.1874454

**Published:** 2026-09-22

**Authors:** Min Min, Wei Zhang, Zhen Zhang

**Affiliations:** 1 School of Supply Chain Management, Ningbo Polytechnic University, Ningbo, China; 2 School of Marxism, Shenzhen Polytechnic University, Shenzhen, China; 3 Business School, Ningbo University, Ningbo, China

**Keywords:** GenAI knowledge seeking, goal orientations, individual creativity, knowledge hiding, new product development

## Abstract

**Purpose:**

Knowledge hiding among colleagues creates problematic information environments that can undermine individual creativity in new product development (NPD) work. While prior research has primarily focused on preventing or reducing knowledge hiding, little is known about how professionals cope with the resulting knowledge gaps in digitally mediated workplaces. Drawing on the attention-based view and achievement goal theory, this study examines whether and when the creativity costs of intra-team knowledge hiding can be partially offset through generative artificial intelligence (GenAI) knowledge seeking.

**Design/methodology/approach:**

We developed and tested a moderated mediation model in which GenAI knowledge seeking mediates the relationship between intra-team knowledge hiding and individual creativity, with goal orientation serving as a moderator. The hypotheses were tested using multi-wave, multi-informant survey data collected from 275 Chinese NPD professionals.

**Findings:**

The results show that intra-team knowledge hiding is negatively associated with individual creativity, but this effect operates through two opposing pathways: a negative direct effect and a positive indirect effect via GenAI knowledge seeking that partially offsets the direct harm. Moreover, learning goal orientation strengthens the positive relationship between knowledge hiding and GenAI knowledge seeking, thereby mitigating the creativity risks associated with colleagues’ knowledge hiding.

**Originality:**

This study advances the literature by broadening attention beyond suppressing knowledge hiding to understanding technology-enabled coping responses. By identifying GenAI knowledge seeking as a compensatory mediating pathway and learning goal orientation as a boundary condition, the study provides novel insights into how AI technologies can help professionals partially sustain creativity under imperfect knowledge-sharing conditions in NPD teams.

## 1 Introduction

To thrive in a competitive market environment, companies must constantly introduce new products and services with novel features (Yam and Chan, 2015). Cross-functional project teams have emerged as a popular organizational structure for boosting new product development (NPD) innovation (Geraldi et al., 2011). Although project professionals are expected to realize their creative potential through sharing common goals and expertise, they are observed sometimes to hide knowledge due to project-based task design and attributes (e.g., scarcity of resources due to goal constraints) (Zhang and Min, 2022; Moh’d et al., 2021). Knowledge hiding has been shown to harm the achievement of seekers’ creative outcomes (Chatterjee et al., 2021; Zhang and Min, 2022) because it disrupts members’ (seekers) access to specialized knowledge (Connelly et al., 2012; Ghasemaghaei and Turel, 2021; Lin et al., 2023). Managing the risks associated with knowledge hiding is crucial for fostering creativity of NPD professionals.

Knowledge hiding within teams refers to colleagues’ deliberate withholding of requested knowledge from seekers (Connelly et al., 2012). Extant research on managing knowledge hiding has primarily explored managerial antecedents that suppress the occurrence of knowledge hiding, such as coordination mechanisms (Zhang and Min, 2022), human resource management (HRM) practices (Oubrich et al., 2021), and leadership styles (Men et al., 2020; Donate et al., 2022). However, in increasingly digitalized work environments, few studies have revealed the digitally enabled response mechanisms through which professionals, as knowledge seekers, respond to knowledge hiding by their teammates during the creativity process.

This research topic is significant for two reasons. First, knowledge hiding is prevalent in project-based work environments and cannot be fully eliminated through managerial antecedents (Zhang and Min, 2024). For instance, despite increasing investments in knowledge management practices (Kankanhalli et al., 2005), knowledge hiding remains a common phenomenon across organizations (Offergelt et al., 2019). Accordingly, examining individuals’ subsequent responses to knowledge hiding is important for understanding and managing the risks associated with knowledge hiding (e.g., knowledge gaps related to tasks).

Second, previous research has primarily focused on seekers’ responses to knowledge hiding from the perspective of internal knowledge transfer, such as reciprocal knowledge hiding from hiders (Serenko and Bontis, 2016), low knowledge contribution and decreased support for hiders (Lin et al., 2023; Connelly and Zweig, 2015). However, these internally focused responses do not restore seekers’ access to the task-relevant knowledge that has been withheld. Knowledge seekers may therefore respond to disrupted internal knowledge access by turning to alternative external sources to fill the resulting knowledge gaps (Santangelo and Phene, 2022; Venkataramani and Tang, 2024).

Recent advances in generative artificial intelligence (GenAI) tools (such as ChatGPT and DeepSeek) allow users to quickly obtain, synthesize, and refine specialized information through natural language interactions (Sanatizadeh et al., 2025). In this study, GenAI knowledge seeking is not treated as an entirely new category of knowledge seeking, but as a distinctive technology-mediated form of external knowledge seeking. Unlike conventional external or online knowledge seeking, which mainly involves retrieving information from people, documents, databases, or online repositories, GenAI knowledge seeking enables iterative interaction and the synthesis of dispersed knowledge into task-specific responses (Sanatizadeh et al., 2025). These features reduce the time and cognitive effort required for external knowledge acquisition (Noy and Zhang, 2023), making GenAI a particularly accessible compensatory channel when internal knowledge sources become unavailable. However, whether knowledge seekers actually turn to GenAI to compensate for disrupted access to internal knowledge remains largely unexplored. This gap highlights the need to examine whether GenAI knowledge seeking constitutes a compensatory response to knowledge hiding and the conditions under which such a response is more likely to occur.

The attention-based view (ABV) provides the primary process explanation for how individuals respond to intra-team knowledge hiding. The ABV posits that organizational and individual actions are shaped by what issues and solutions decision-makers focus their attention on (Ocasio, 1997). In NPD work settings, individuals possess limited attentional capacity and must selectively allocate attention toward information sources that are accessible and relevant to creative task demands (Ocasio et al., 2018). Under conditions of intra-team knowledge hiding, the availability of internal knowledge sources is disrupted (Connelly et al., 2012; Connelly and Zweig, 2015). Consequently, knowledge seekers may redirect their attention away from inaccessible internal sources toward alternative knowledge channels that can compensate for emerging knowledge gaps. In digitally mediated work environments, GenAI tools provide a salient and accessible channel for acquiring task-related knowledge (Sanatizadeh et al., 2025; Noy and Zhang, 2023). From an ABV perspective, the distinctive accessibility and interactive synthesis provided by GenAI make it a salient attentional target when intra-team knowledge flows are disrupted. GenAI knowledge seeking may therefore constitute a positive indirect pathway linking intra-team knowledge hiding and individual creativity.

However, the ABV alone does not fully explain why individuals exposed to the same knowledge-hiding condition differ in the extent to which they engage in GenAI knowledge seeking. To explain this variation, we draw on achievement goal theory (AGT), which specifies how individuals’ motivational orientations shape their interpretations of and responses to achievement-related situations (Dweck and Leggett, 1988). Goal orientation refers to the cognitive framework through which individuals perceive and react to achievement situations. When experiencing colleagues’ knowledge hiding, individuals with different goal orientations may form distinct evaluations of the learning opportunities, performance benefits, and potential risks associated with using GenAI to acquire missing knowledge. For instance, individuals with a strong learning goal orientation tend to enjoy challenges and value solution-oriented effort, and they may therefore view GenAI knowledge seeking as an effective response to knowledge hiding. By contrast, individuals who emphasize avoiding unfavorable evaluations may be more sensitive to the uncertainty and possible inaccuracy of AI-generated knowledge.

Although the two perspectives differ in their starting points, they are complementary rather than redundant. ABV locates the impetus for attentional reallocation in external, situational disruption (Ocasio et al., 2018), whereas AGT treats goal orientation as a relatively stable, internally driven disposition (Dweck and Leggett, 1988; VandeWalle, 1997). This very difference is what makes the two theories complementary: the externally triggered attentional shift specified by ABV does not translate uniformly into behavior, and AGT supplies the internal motivational filter that determines for whom, and how strongly, that shift is enacted (Payne et al., 2007). In this sense, ABV explains the general attentional process through which knowledge hiding redirects seekers toward GenAI, whereas AGT identifies the motivational conditions under which that attentional shift is more or less strongly translated into actual knowledge-seeking behavior.

Accordingly, this study attempts to understand: (1) whether GenAI knowledge seeking mediates the relationship between intra-team knowledge hiding and individual creativity, and (2) whether goal orientations intervene in this mediation by moderating the direct effect of knowledge hiding on GenAI knowledge seeking.

The current study appears to make significant contributions to the existing literature on knowledge management in several ways. First, it broadens attention beyond suppressing knowledge hiding to understanding how knowledge seekers compensate for disrupted internal knowledge access through a distinctive technology-mediated form of external knowledge seeking. By emphasizing GenAI’s conversational interaction, knowledge synthesis, and relatively low search costs, this study clarifies why GenAI knowledge seeking represents more than the simple use of another external information source. Second, drawing on the ABV and AGT, this study develops a process-oriented and contingent explanation of how knowledge hiding influences individual creativity. The ABV explains the attentional mechanism linking knowledge hiding to GenAI knowledge seeking, whereas AGT explains the individual-level boundary conditions that strengthen or weaken this mechanism. Third, despite the well-documented creativity barriers associated with knowledge hiding, the study identifies a partial compensatory pathway through which some of these barriers may be offset, providing empirical evidence for a more complete understanding of the consequences of knowledge hiding within NPD projects.

## 2 Literature review and hypothesis development

### 2.1 Intra-team knowledge hiding as a barrier for NPD professionals’ creativity

As stated earlier, cross-functional teams bring multidisciplinary knowledge into the NPD processes, which creates opportunities for project professionals to generate novel ideas and unique solutions (Nakata et al., 2018). However, these benefits can be only realized when professionals are aware of their teammates’ expertise and obtain essential knowledge from them (Tang et al., 2015). Nevertheless, many project professionals find that their teammates do not always satisfy their knowledge requests and sometimes prefer to hide knowledge from them (Zhang and Min, 2022). The attributes of NPD projects, such as temporariness and cross-functionality, may serve as an inherent reason for the occurrence of knowledge hiding.

For example, NPD professionals from different functional departments are temporarily assembled to achieve challenging project goals with limited work resources (Weiss et al., 2011; Edmondson and Nembhard, 2009). Team members would contest for confined resources and power during NPD endeavors, leading to cross-functional competition within projects (Luo et al., 2006). As a result, this team competition further results in knowledge transfer barriers and interpersonal knowledge hiding (Oubrich et al., 2021). Moreover, to achieve the challenging project goals, project professionals need to sacrifice individual resources (e.g., extra time and energy) and experience psychological stress, which motivates them to engage in hiding knowledge as a stress-coping approach (Venz and Nesher Shoshan, 2022). As new product projects are increasingly complex and challenging, knowledge hiding behaviors have become a prevalent phenomenon (Zhang and Min, 2024).

Given that knowledge hiding restricts seekers’ access to relevant information and is intentional, dyadic, and counterproductive in nature (Connelly and Zweig, 2015; Connelly et al., 2012), scholars have identified its potential influence on creativity in innovative project settings. Creativity refers to the generation of novel and potentially useful ideas, solutions, or processes for an organization (Amabile, 1988; Madjar et al., 2011). For example, knowledge hiding was found to interrupt interpersonal trust among project professionals, demotivate knowledge seekers to collaborate with hiders, and be harmful to individual and organizational creative outcomes (Zhang and Min, 2022; Chatterjee et al., 2021). Moreover, intra-team knowledge hiding prevents professionals, as knowledge seekers, from obtaining diverse and relevant perspectives, thereby hindering their ability to generate new ideas and solutions by considering multiple possibilities (Bogilović et al., 2017; Černe et al., 2014). Consequently, knowledge hiding among colleagues is considered detrimental to the creativity of NPD professionals. Given this consistent evidence, we treat the negative direct effect of intra-team knowledge hiding on creativity as a theoretically anticipated relationship in our model, alongside its hypothesized indirect, compensatory effect through GenAI knowledge seeking.

Specifically, knowledge hiding is conceptualized as a multidimensional construct that includes evasive hiding (e.g., providing false information), playing dumb (e.g., pretending not to know the knowledge), and rationalized hiding (e.g., rejection with rationale excuses) (Connelly et al., 2012). Recent studies have emphasized that rationalized hiding may arise from legitimate or ethical considerations, such as confidentiality requirements, and that knowledge withheld for such reasons may be less relevant or appropriate for transfer (Ghasemaghaei and Turel, 2021). In contrast, evasive hiding and playing dumb involve misleading the knowledge seeker or concealing possession of the requested knowledge. Accordingly, rather than examining general intra-team knowledge hiding in its broader three-dimensional form, the current study focuses specifically on deceptive intra-team knowledge hiding, operationalized through evasive hiding and playing dumb. These two forms more clearly indicate that the internal knowledge source is unwilling or unreliable, thereby prompting knowledge seekers to consider alternative knowledge channels. For brevity, we use the term intra-team knowledge hiding throughout the manuscript to refer to this deceptive form.

Considering the negative effect of knowledge hiding on creativity, great efforts have been made to identify managerial practices for managing knowledge hiding (Oubrich et al., 2021; Donate et al., 2022; Men et al., 2020). However, knowledge hiding is inevitable and may not be completely removed by managerial practices due to NPD projects’ temporariness and cross-functionality. Existing findings on seekers’ reaction towards perpetrators’ knowledge hiding are limited to mechanisms that mediate the negative performance effects of knowledge hiding (Lin et al., 2023; Serenko and Bontis, 2016). The current study focuses on ex post coping approaches (seekers’ responses) that transmit the possible positive effect of knowledge hiding on NPD professionals’ creativity. Consistent with prior research, we examine intra-team knowledge hiding as perceived by the knowledge seeker, given that it is the seeker’s perception of being subjected to knowledge hiding within the team that shapes the seeker’s subsequent reactions (Černe et al., 2014).

### 2.2 GenAI knowledge seeking as a mediator

The core reason why knowledge hiding undermines creativity in NPD projects lies in its obstruction of knowledge seekers’ access to task-relevant information and expertise (Zhang and Min, 2024). When colleagues deliberately withhold requested knowledge, internal knowledge-seeking channels become unreliable, leaving seekers with unmet informational needs during creative problem solving and idea generation (Zhang and Min, 2022). In such situations, individuals may respond by reducing effort or by continuing to search for knowledge through colleagues, external experts, organizational databases, or other sources. Because NPD tasks are often interdependent and time-sensitive, reducing effort may leave the immediate knowledge gap unresolved and impede task progress. Knowledge seekers are therefore more likely to continue searching through alternative channels that can provide the knowledge needed to address the focal problem.

According to the ABV, individuals do not respond automatically to environmental constraints; instead, they selectively allocate attention to information sources that are perceived as most viable for addressing salient task demands (Ocasio et al., 2018). In the context of intra-team knowledge hiding, the originally preferred interpersonal knowledge source becomes less reliable, prompting individuals to redirect their attention toward alternative channels that are perceived as both accessible and capable of addressing the immediate knowledge gap. Within this attentional reconfiguration process, GenAI knowledge seeking, which refers to external knowledge sourcing that is supported by GenAI technology (Zhang et al., 2023), represents one particularly salient alternative. Compared with repeated interpersonal searches or static knowledge repositories, GenAI tools enable individuals to rapidly retrieve, synthesize, and refine task-relevant information through interactive, natural language–based queries (Sanatizadeh et al., 2025). As a result, when internal interpersonal knowledge channels become unreliable while task demands remain unchanged, engaging in GenAI knowledge seeking reflects an intentional redirection of attention toward an immediately accessible, technology-enabled knowledge channel that can supplement disrupted intra-team knowledge flows. Accordingly, intra-team knowledge hiding is expected to increase individuals’ engagement in GenAI knowledge seeking as a means of compensating for internal knowledge deficiencies.

Although knowledge hiding may elicit various responses, including reciprocal hiding, reduced knowledge contribution, and decreased support for the hider (Serenko and Bontis, 2016; Connelly and Zweig, 2015; Lin et al., 2023), it leaves knowledge seekers with unresolved informational needs that must still be addressed to advance their tasks (Zhang and Min, 2022, 2024). From an attention-based perspective, the reduced viability of the original internal knowledge source increases the salience of alternative channels that can help restore informational continuity (Ocasio, 1997; Ocasio et al., 2018). Cross-team colleagues and external experts remain possible alternatives, yet their availability depends on others’ willingness and the social cost of asking, and turning to them reintroduces the very interpersonal barriers that knowledge hiding has already raised (Connelly et al., 2012; Serenko and Bontis, 2016). By contrast, GenAI is asynchronously and privately accessible, imposes no reciprocal obligation, and returns synthesized rather than fragmented responses (Noy and Zhang, 2023; Sanatizadeh et al., 2025). We therefore do not claim that GenAI dominates these alternatives on every dimension (Santangelo and Phene, 2022; Venkataramani and Tang, 2024); rather, its comparatively low interpersonal and temporal cost makes it an especially salient attentional target when internal knowledge access is disrupted. Accordingly, intra-team knowledge hiding is expected to increase individuals’ engagement in GenAI knowledge seeking. We propose the following:

*Hypothesis 1*: Intra-team (perceived colleagues’) knowledge hiding is positively related to GenAI knowledge seeking.

Knowledge seeking is an activity oriented toward acquiring task-relevant knowledge, and it can be enacted through different channels, including seeking knowledge from others, consulting codified sources, seeking through digital platforms, or using GenAI (Cross and Sproull, 2004; Choo, 1996; Mickeler et al., 2023). A seeker must choose among these channels according to their accessibility and cost. GenAI knowledge seeking is therefore one form of knowledge seeking rather than a substitute for the activity itself, and being channel-specific is common to all seeking rather than particular to GenAI. What matters is how a channel reshapes the activity: through its interactivity, privacy, and low cost, GenAI reshapes how task-relevant knowledge is acquired and initially synthesized (Jarrahi et al., 2023; Wang et al., 2025). The interactive nature of GenAI lets the seeker probe and question responses, supporting the evaluation of whether the knowledge fits the focal problem. Its capacity to synthesize across sources supports recombination, connecting the retrieved knowledge to the task at hand. The recombined knowledge can then be applied to creative problem solving, thereby supporting creativity (Cohen and Levinthal, 1990; Nonaka, 1994; Zahra and George, 2002).

Intra-team knowledge hiding may therefore influence creativity through two opposing pathways. On the one hand, it directly restricts access to context-specific and relational knowledge embedded within the team and may weaken trust and subsequent collaboration, thereby undermining creativity (Bogilović et al., 2017; Černe et al., 2014; Connelly and Zweig, 2015; Zhang and Min, 2022). On the other hand, the resulting knowledge deficiency may redirect individuals’ attention toward GenAI-enabled external knowledge sources.

When this attentional shift leads to GenAI knowledge seeking, professionals may obtain alternative information that broadens their understanding of the problem and supports creative exploration. Prior research suggests that GenAI-assisted solution search can expand the range of potential solutions and improve creative problem-solving outcomes (Boussioux et al., 2024). Although some GenAI-generated information may be insufficiently aligned with the focal context, it can still provide useful knowledge for considering possible solutions. This evidence suggests that GenAI can provide useful support in knowledge-intensive work, although the extent of its benefits may vary across task contexts (Dell’Acqua et al., 2026). Thus, GenAI knowledge seeking provides a partial compensatory pathway through which intra-team knowledge hiding may have a positive indirect effect on creativity despite its direct negative consequences. Therefore, we propose the following:

*Hypothesis 2*: Intra-team knowledge hiding has a positive indirect effect on a professional’s creativity through GenAI knowledge seeking.

### 2.3 Goal orientation as contingencies

According to AGT, goal orientation reflects individuals’ inherent or situational preferences in achievement contexts, which may lead professionals to form distinct cognitive evaluations regarding how to respond to task demands (e.g., information gaps resulting from knowledge hiding) (Brett and VandeWalle, 1999; LePine, 2005; VandeWalle, 1997). Drawing on this perspective, different types of goal orientation can be viewed as contingencies that intervene in individuals’ judgments about the effectiveness of GenAI knowledge seeking as a response to knowledge hiding.

Goal orientation is commonly divided into learning goal orientation and performance goal orientation, with the latter further categorized into performance-prove and performance-avoid orientations (Brett and VandeWalle, 1999). Specifically, learning goal orientation emphasizes the acquisition of knowledge and the improvement of skills and abilities (Payne et al., 2007; Hirst et al., 2011). When knowledge hiding occurs, an NPD professional with a high level of learning goal orientation places greater emphasis on continuous learning and shows stronger motivation to seek information that cannot be obtained within the team. GenAI knowledge seeking provides access to diverse external knowledge and learning opportunities, which are particularly valuable for self-development and creative problem solving (Tang, 2016). Therefore, learning-oriented individuals who experience intra-team knowledge hiding are more likely to engage in GenAI knowledge seeking.

Performance-prove goal orientation reflects individuals’ desire to demonstrate competence and obtain favorable judgments from others (Payne et al., 2007). When performance-prove orientation is high, professionals whose access to the knowledge required for task accomplishment is constrained by intra-team knowledge hiding may become particularly concerned about maintaining strong task performance through which their competence can be demonstrated. GenAI knowledge seeking provides an alternative means of obtaining task-relevant information when the needed knowledge cannot be accessed from colleagues (Singh et al., 2018). Because this search can be conducted privately without directly revealing one’s knowledge deficiency to other team members, it may help performance-prove-oriented professionals address the knowledge gap while limiting threats to their desired image of competence. By supporting task performance, GenAI knowledge seeking may therefore help them secure favorable evaluations. Accordingly, performance-prove goal orientation is expected to strengthen the positive relationship between intra-team knowledge hiding and GenAI knowledge seeking.

In contrast, performance-avoid goal orientation centers on avoiding mistakes, unfavorable evaluations, and negative performance outcomes (Payne et al., 2007). Avoidance-oriented professionals tend to focus on potential risks when selecting response strategies under uncertainty. Although GenAI knowledge seeking can provide abundant information, professionals may face uncertainty about whether its outputs are appropriate for the focal task. Given their strong concern with avoiding mistakes and negative evaluations, performance-avoid-oriented professionals are more likely to view this uncertainty as increasing the risk of unfavorable task outcomes (Rivkin, 2000). Consequently, even when intra-team knowledge hiding creates a need for alternative knowledge, they may be reluctant to rely on GenAI, thereby weakening the positive relationship between intra-team knowledge hiding and GenAI knowledge seeking. Taken together, we propose the following:

*Hypotheses 3a, 3b, and 3c*: Learning goal orientation (a) and performance-prove goal orientation (b) positively moderate (strengthen) the positive effect of intra-team knowledge hiding on GenAI knowledge seeking; in contrast, performance-avoid goal orientation (c) negatively moderates (weakens) this effect.

Building on ABV, we propose that intra-team knowledge hiding influences creativity indirectly through GenAI knowledge seeking. Accordingly, goal orientation may further shape the strength of this indirect relationship by influencing the extent to which knowledge hiding elicits GenAI knowledge seeking. When learning or performance-prove goal orientation strengthens the relationship between deceptive intra-team knowledge hiding and GenAI knowledge seeking, the same level of knowledge hiding is more likely to generate greater engagement in GenAI knowledge seeking, thereby producing a stronger positive indirect effect on creativity. In contrast, when performance-avoid goal orientation weakens this relationship, less GenAI knowledge seeking is elicited, resulting in a weaker indirect effect. Thus, the three forms of goal orientation are expected to shape the indirect effect of deceptive intra-team knowledge hiding on creativity by altering the strength of its first-stage relationship with GenAI knowledge seeking. Therefore, we propose the following:

*Hypotheses 4a, 4b, and 4c*: The indirect effect of intra-team knowledge hiding on creativity through GenAI knowledge seeking is positively moderated (strengthened) by learning goal orientation (a) and performance-prove goal orientation (b); in contrast, performance-avoid goal orientation (c) negatively moderates (weakens) this indirect effect.

## 3 Methodology

### 3.1 Sample and data collection

Digitalization has revolutionized the way we innovate by introducing innovative smart products and services (Wetzels, 2021). This motivated us to engage computer science and technology experts involved in research and development (R&D) work as potential informants. The selection of participants was facilitated by an alumni association at a prominent university in eastern China. We targeted alumni who had a degree in computer science and held positions as directors and engineers within new product projects. These individuals displayed a keen interest in our research topic and were invited to participate in our survey. To ensure diverse perspectives and prevent reliance on a single source of information, we also invited their supervisors to take part in the survey. In total, we collected responses from 412 R&D professionals. To ensure that the survey was conducted in an ethical manner, participation was voluntary. Before completing the questionnaire, all participants were informed of the academic purpose of the study and provided informed consent. Responses were collected anonymously, and the study was conducted in accordance with ethical standards for research involving human participants.

In mainland China, we conducted an empirical survey. Since the measurement scales used for the research variables were initially developed in English, we followed the widely used back-translation procedure (Brislin, 1970). The original English version of each scale was translated into Chinese by one translator, and then another translator back-translated the Chinese version into English, ensuring the equivalence of meaning. To validate the feasibility of the translated scales, we conducted a pilot study involving four R&D managers and their subordinates. The translated scales accurately and completely expressed the intended meanings, indicating their suitability for data collection.

Consistent with prior research emphasizing the need for self-reported approaches in data collection, particularly for studying knowledge hiding (Connelly et al., 2012; Zhang and Min, 2024), we employed a multiple-informant and time-lagged online survey (Park and Lee, 2014; Lin et al., 2015). This approach helps address potential problems of common method bias (CMB), such as leniency biases, social desirability, and transient mood states (Podsakoff et al., 2003). To ensure proper matching of questionnaires between professionals and their respective supervisors, we assigned similar codes to paired questionnaires. According to prior research on knowledge hiding (Arain et al., 2020a; Arain et al., 2020b), selecting two or fewer members per team can enhance the reliability of individual creativity ratings while reducing within-group variance. In line with our research focus, this sampling approach allowed us to capture members’ responses to differentiated intra-team knowledge hiding.

To mitigate concerns about common method bias, we adopted a three-wave survey design with a two-week interval between waves and collected data from multiple sources, including project managers and team members (Podsakoff et al., 2003; Zhu et al., 2019). Of the 412 employees initially invited, 367 completed the first-wave survey, in which they rated their perceived intra-team knowledge hiding and goal orientation (learning, proving, and avoidance). In the second wave, conducted 2 weeks later, the same employees rated their GenAI knowledge seeking, yielding 311 valid responses. In the third wave, their corresponding supervisors evaluated the creativity of each employee. To increase response rates, we sent reminder emails after 2 weeks and made follow-up telephone calls after 4 weeks. After excluding responses with incomplete matching information or missing data, the final sample comprised 275 professionals from 87 organizations and 167 project teams, with creativity ratings provided by 167 supervisors. Each team contributed no more than two employee respondents: 108 teams contributed two and 59 contributed one, yielding an average cluster size of 1.647. Information on the informants and their project teams is presented in Table 1.

**Table 1:** The profiles of participants (*N* = 275).

Item	Number	Percent
Gender		
Male	214	77.8%
Female	61	22.2%
Age		
25 years or less	55	20.0%
26–30 years	68	24.7%
31–35 years	60	21.8%
36–40 years	43	15.6%
41 years or more	49	17.8%
Education		
Bachelor’s degree	81	29.5%
Master’s degree	162	58.9%
Doctoral degree	32	11.6%
Team tenure		
4 months or less	82	29.8%
5–8 months	81	29.5%
9–12 months	70	25.5%
13 months or more	42	15.2%
Project stage		
Predevelopment	85	30.9%
Develop and commercialization	190	69.1%
Industrial type		
Manufacturing	104	37.8%
Software	73	26.5%
Telecommunication	47	17.1%
Others	51	18.5%

### 3.2 Measures

In the current study, all items were rated on a seven-point Likert-type scale ranging from 1 (strongly disagree) to 7 (strongly agree).

#### 3.2.1 Intra-team knowledge hiding

The 12-item knowledge hiding scale developed by Connelly et al. (2012) has been widely used to capture perpetrators’ different strategies of evasive hiding (four items), playing dumb (four items), and rationalized hiding (four items). Different from rationalized hiding, evasive hiding and playing dumb are characterized by deceptive intentions and are more likely to provoke retaliatory or active coping behaviors from seekers when they perceive these behaviors (Connelly and Zweig, 2015; Zhang and Min, 2024). To eliminate interference resulting from inconsistent natures and difference in individuals’ perceptions, the current study mainly focused on deceptive intra-team knowledge hiding that was perceived by professionals and examined this variable at the individual level. A simplified 8-item scale including evasive hiding and playing dumb was further adopted. The use of this composite measure was further supported by additional exploratory and confirmatory factor analyses: a correlated two-factor model distinguishing evasive hiding from playing dumb showed numerically better fit but the latent factor covariance matrix was not positive definite (factor correlation > 1 under both MLR and WLSMV estimation), indicating that the two dimensions could not be reliably distinguished in this sample, whereas a single-factor model and an exploratory factor analysis both supported a dominant one-factor structure (EFA first factor explaining 80.43% of variance). All the items started with a scenario: ‘During your project, at a time when you requested knowledge from your teammates and they refused’.

#### 3.2.2 Goal orientation

Goal orientation can be classified into three categories: learning, performance-avoid, and performance-prove goals (Brett and VandeWalle, 1999). The scale developed by VandeWalle (1997) was used to measure these three types of goal orientation. Five items were employed to measure learning goal orientation, four items were utilized to measure performance-avoid goal orientation, and another four items were used to measure performance-prove goal orientation.

GenAI knowledge seeking. A 3-item scale adapted by Zhang et al. (2023) for R&D settings was used for measuring NPD professionals’ AI-enabled knowledge seeking. Informants were requested to respond to the extent to which they frequently use GenAI tools (e.g., DeepSeek and ChatGPT) for seeking external knowledge outside their projects.

#### 3.2.3 Creativity

The six-item creativity scale developed by Madjar et al. (2011) was employed to capture NPD professionals’ creativity. The scale includes items originally designed to assess adaptive and radical creativity. Although the scale includes these two types of creativity items, we conceptualize creativity as an overall construct reflecting employees’ ability to generate novel and useful ideas, consistent with prior research in R&D contexts (Tang, 2016). To empirically verify this measurement decision, we conducted an exploratory factor analysis (EFA) of the six creativity items. The results revealed a single-factor solution, with the first factor having an eigenvalue of 4.820 and explaining 80.3% of the total variance. The second component had an eigenvalue of only 0.438. These results provide support for treating creativity as a unidimensional construct in this study.

#### 3.2.4 Control variables

Consistent with prior research on creative performance among project professionals, individual demographic characteristics (i.e., gender, age, education, and team tenure), as well as team size, may influence our core variables and are thus treated as control variables (Tang, 2016; Hirst et al., 2011). Age (in years), team tenure (in months), and team size (number of members) were coded as continuous variables. Degree of education was marked as an ordinal variable (1 = bachelor’s degree, 2 = master’s degree, and 3 = doctoral degree). Additionally, the fuzzy front-end phase marks the beginning of opportunity ideation, exploration, and assessment, and it relies heavily on individual creativity (Thanasopon et al., 2016; Blauth et al., 2014). The different focus of work between the front-end and other phases may result in varying levels of effort towards creativity. Therefore, we also controlled for project phase (0 = fuzzy front-end; 1 = development and commercialization phases).

### 3.3 Non-response bias

To identify any potential issues arising from non-response bias, we applied the time-trend extrapolation method (Armstrong and Overton, 1977). Following this procedure, an independent-samples t-test was conducted to compare the main variables between early respondents (the first quartile of returned surveys, *N* = 69) and late respondents (the last quartile, *N* = 68). The t-values for intra-team knowledge hiding, GenAI knowledge seeking, creativity, and the three types of goal orientation ranged from 0.100 (n.s.) to 0.536 (n.s.), with all *p*-values exceeding 0.59, indicating no significant differences in any of the research variables. These results provide no evidence of non-response bias in the current sample.

### 3.4 Common method bias

To address common method bias, as suggested by Podsakoff et al. (2003), we employed a multiple-informant, multiple-wave design: employees self-reported intra-team knowledge hiding, goal orientation, and GenAI knowledge seeking across three waves spaced 2 weeks apart, while their supervisors independently rated creativity. This separation of sources and time points helps attenuate common method bias. As a preliminary screen, we conducted Harman’s single-factor test; it returned six factors with eigenvalues above 1.0, with no single factor accounting for more than 27.3% of the variance, below the 50% cutoff. Because passing this test is necessary but not sufficient, we further applied the latent method factor control recommended by Liang et al. (2007). The average substantively explained variance of the indicators was 0.765, whereas the average method-based variance was 0.003 (a ratio of approximately 250:1), and none of the method-factor loadings were significant. Together, these results indicate that common method bias is not a serious concern in this study.

### 3.5 Analysis method

In this study, our focus was on exploring the sequential relationships between perceived intra-team knowledge hiding, GenAI knowledge seeking, and individual creativity, as well as the moderating roles of goal orientation at the individual level, rather than at the multilevel or team level. To effectively test complex moderating and mediating effects, specifically moderated mediation, we employed Mplus software version 8.3 (Muthén and Muthén, 1998–2019) as our analytical approach. Mplus is widely recognized and utilized in individual-level analysis studies for its ability to handle intricate mediating relationships. To assess the significance of the hypothesized direct and indirect effects, we constructed 95% confidence intervals (CIs) and applied two-tailed criteria. Additionally, we conducted bootstrapping with 10,000 steps to ensure the robustness of our analyses. The structural model was estimated using observed composite variables. Interaction terms were created as products of grand-mean-centered predictors (intra-team knowledge hiding × each goal orientation), and conditional indirect and total effects were computed through the MODEL CONSTRAINT command with bias-corrected bootstrap confidence intervals.

## 4 Results

### 4.1 Reliability and validity

The details of factor loadings, Cronbach’s alpha coefficients, composite reliability (CR) and average variance extracted (AVE) are reported in Table 2. Specifically, the Cronbach’s alphas and CR values of each variable exceeded the recommended threshold of 0.7 (Bagozzi and Yi, 1988), indicating accepted construct reliability. To evaluate the construct validity of the adopted scales, confirmatory factor analyses (CFA) were conducted for the research variables (i.e., knowledge hiding, goal orientation, GenAI knowledge seeking, creativity). The factor loadings of each measurement indicator ranged from 0.705 to 0.961, which exceeded the suggested threshold of 0.7. The AVEs for the six research variables ranged from 0.579 to 0.790, which surpassed the recommended level of 0.5. The evidence supported satisfactory convergent validity for each construct.

**Table 2:** The indices for construct reliability and convergent validity.

Construct/item	Factor loading	Cronbach’s alpha	CR	AVE
Intra-team knowledge hiding (ITKH) *(responded by professionals)*		0.969	0.959	0.744
During your project, at a time when you requested knowledge from your teammates and they refused:				
*Evasive hiding*				
ITKH1: My teammates promised to provide me the requested information but never really intended to.	0.748			
ITKH2: My teammates promised to provide me the requested information but instead gave me information different from what I wanted.	0.950			
ITKH3: My teammates told me that he/she would help me later but postponed as long as possible.	0.812			
ITKH4: My teammates offered me some other information than what I really wanted.	0.961			
*Playing dumb*				
ITKH5: My teammates pretended that he/she did not have the requested information.	0.764			
ITKH6: My teammates said that he/she did not know, although he/she did.	0.888			
ITKH7: My teammates pretended he/she did not know what I was talking about.	0.878			
ITKH8: My teammates said that he/she was not familiar with the subject.	0.875			
Goal orientation *(responded by professionals)*				
*Learning goal (LG)*		0.895	0.896	0.633
LG1: I am willing to select a challenging work assignment that I can learn a lot from.	0.836			
LG2: I often look for opportunities to develop new skills and knowledge.	0.810			
LG3: I enjoy challenging and difficult tasks at work where I’ll learn new skills.	0.760			
LG4: For me, development of my work ability is important enough to take risks.	0.765			
LG5: I prefer to work in situations that require a high level of ability and talent.	0.803			
*Performance avoid goal (PAG)*		0.843	0.846	0.579
PAG1: I would avoid taking on a new task if there was a chance that I would appear rather incompetent to others.	0.705			
PAG2: Avoiding a show of low ability is more important to me than learning a new skill.	0.784			
PAG3: I’m concerned about taking on a task at work if my performance would reveal that I had low ability.	0.816			
PAG4: I prefer to avoid situations at work where I might perform poorly.	0.735			
*Performance prove goal (PPG)*		0.856	0.857	0.599
PPG1: I like to show that I can perform better than my coworkers.	0.787			
PPG2: I try to figure out what it takes to prove my ability to others at work.	0.748			
PPG3: I enjoy it when others at work are aware of how well I am doing.	0.796			
PPG4: I prefer to work on projects where I can prove my ability to others.	0.766			
Generative AI knowledge seeking (GAIKS) *(responded by professionals)*		0.910	0.919	0.790
GAIKS1: In this project, I often use Generative Artificial Intelligence tools to seek knowledge for tasks.	0.876			
GAIKS2: In this project, I frequently use Generative Artificial Intelligence tools to seek knowledge relevant to tasks.	0.889			
GAIKS3: In this project, I spend a lot of time using Generative Artificial Intelligence tools to seek knowledge essential for tasks.	0.901			
Creativity (C) *(responded by supervisors)*		0.950	0.945	0.742
Please evaluate your (the target) subordinate’s creativity within your project:				
C1: My subordinate is a good source of highly creative ideas and solutions.	0.776			
C2: My subordinate demonstrates originality in his/her work.	0.892			
C3: My subordinate suggests radically new ways for doing work.	0.911			
C4: My subordinate is very good at adapting already-existing ideas.	0.882			
C5: My subordinate easily modifies previously existing work processes to suit current needs.	0.838			
C6: My subordinate uses previously existing ideas or work in an appropriate new way.	0.861			

Drawing on the Fornell-Larcker criterion, we evaluated the discriminant validity for studied variables. Square roots of the AVEs for each variable were significantly greater than the corresponding cross-correlations (see Table 3). In addition, we computed heterotrait-monotrait (HTMT) ratios of correlations, which provide a more stringent assessment of discriminant validity (Henseler et al., 2015). All HTMT values fell well below the conservative threshold of 0.85, with the highest ratio observed between knowledge hiding and GenAI knowledge seeking (0.479), followed by learning goal orientation and GenAI knowledge seeking (0.469). Among the three goal orientations, HTMT ratios ranged from 0.163 (learning and prove) to 0.395 (prove and avoid), confirming that these conceptually proximate constructs are empirically distinct. Moreover, we also performed alternative CFA models (e.g., five-factor and four-factor constructs), the original six-factor construct (i.e., knowledge hiding, GenAI knowledge seeking, creativity, learning, performance-prove, performance-avoid goal orientation) yielded the greatest model fit values [*χ*^2^ (385) = 1144.160, *p* < 0.01; CFI = 0.94, TLI = 0.93, SRMR = 0.05, RMSEA = 0.07], implying good discriminant validity.

**Table 3:** The indices for discriminant validation.

Constructs	M	SD	1	2	3	4	5	6	7	8	9	10	11	12
1. Gender	0.22	0.42	–											
2. Age	32.27	6.74	0.107	–										
3. Education	1.82	0.62	−0.073	0.129*	–									
4. Team tenure	7.77	4.15	0.008	−0.050	−0.103	–								
5. Product phase	0.69	0.46	0.054	0.045	−0.053	0.131*	–							
6. Team size	9.42	2.64	−0.035	−0.051	−0.093	0.058	0.025							
7. ITKH	4.06	1.08	−0.048	−0.006	−0.062	−0.068	0.060	0.044	**0.863**					
8. LG	4.75	0.86	0.074	0.106	0.049	−0.137*	0.108	−0.142	0.001	**0.796**				
9. PPG	4.63	0.58	−0.040	0.077	0.071	−0.068	−0.022	0.002	−0.033	0.144*	**0.761**			
10. PAG	3.35	0.57	−0.024	0.052	−0.071	0.041	−0.005	0.095	−0.070	−0.299**	−0.330**	**0.774**		
11. GAIKS	4.53	0.88	−0.042	0.090	0.102	−0.217**	0.094	−0.154*	0.439**	0.416**	−0.086	−0.071	**0.889**	
12. Creativity	5.24	1.12	0.048	0.247**	0.018	0.012	0.091	0.024	−0.169**	0.231**	0.005	−0.032	0.202**	**0.861**

Diagonal elements in bold font show the square root of AVE for the corresponding construct. M denotes mean; SD denotes standard deviation.

ITKH, intra-team knowledge hiding; LG, learning goal; PPG, performance-prove goal; PAG, performance-avoid goal; GAIKS, Generative Artificial Intelligence knowledge seeking.

*p < 0.05, **p < 0.01; *N* = 275.

Although all constructs were specified at the individual level, we empirically assessed whether the nested structure of respondents within project teams affected our estimates. A two-level null model yielded ICC(1) values of 0.080, 0.092, and 0.018 for intra-team knowledge hiding, GenAI knowledge seeking, and creativity, respectively, with corresponding ICC(2) values of 0.125, 0.143, and 0.029, all below the 0.70 threshold for reliable group-level aggregation (LeBreton and Senter, 2008). Given an average cluster size of 1.647, the resulting design effects (1.012–1.059) suggest that conventional individual-level standard errors may be underestimated by no more than 2.9%. We further re-estimated the model using project-team clustered standard errors; point estimates remained virtually unchanged, and all hypothesized paths retained the same direction and significance levels. Between-team variance was not statistically distinguishable from zero for any construct (all *p* > 0.33), and a parallel model using cluster-robust standard errors (TYPE = COMPLEX) produced standard-error increases of at most 5.1%, confirming that clustering does not materially affect inference here. Full cluster-robust estimates for all focal paths are reported in Appendix A.

### 4.2 Hypothesis testing and key findings

All coefficients reported in the text and Tables 4, 5 are unstandardized path estimates. We report unstandardized estimates because the conditional indirect and total effects in Table 5 were computed from unstandardized structural parameters using the MODEL CONSTRAINT command. The structural model demonstrated good fit to the data [*χ*^2^(6) = 7.749; RMSEA = 0.033; CFI = 0.991; TLI = 0.951; SRMR = 0.020]. Three SEM models were built for hypothesis testing. In the first model, the total effect of intra-team knowledge hiding on creativity was examined. In the second model, the relationship between intra-team knowledge hiding and GenAI knowledge seeking, as well as the moderating effects of three types of goal orientation were computed. In the third model, the mediating role of GenAI in the relationship between intra-team knowledge hiding and creativity as well as the moderating roles of three types of goal orientation in the mediation relationship were examined. The outputs for the three models are described in Figure 1 and Table 4.

**Table 4:** Path modeling results.

Paths	Model 1 dependent variable: creativity	Model 2 dependent variable: GenAIKS	Model 3 dependent variable: creativity
Direct path			
Gender	0.045	−0.153	0.074
Age	0.040*	0.004	0.036*
Education	0.012	0.073	−0.010
Team tenure	−0.001	−0.020	0.016
product phase	0.215	0.076	0.136
Team size	0.018	−0.030	0.040
Software industry	−0.118	−0.060	−0.072
Telecommunication industry	−0.005	0.069	−0.019
Other industries	−0.001	−0.082	0.034
Intra-team knowledge hiding (ITKH)	−0.182*	0.374** *H1 supported*	−0.328**
Learning goal (LG)		0.459**	
Performance-prove goal (PPG)		−0.086	
Performance-avoid goal (PAG)		0.117	
ITKH*LG		0.135* *H3a supported*	
ITKH*PPG		0.044 *H3b rejected*	
ITKH*PAG		−0.076 *H3c rejected*	
GenAI knowledge seeking (GenAIKS)			0.427**
Conditional indirect path			
ITKH → GenAIKS → Creativity			0.160** *H2 supported*
ITKH*LG → GenAIKS → Creativity			0.058* *H4a supported*
ITKH*PPG → GenAIKS → Creativity			0.019 *H4b rejected*
ITKH*PAG → GenAIKS → Creativity			−0.033 *H4c rejected*

Manufacturing was the reference category, with dummy variables created for software industry, telecommunication industry, and others. Coefficients are unstandardized path estimates.

N = 275, *p < 0.05, **p < 0.01.

**Table 5:** Conditional total effects of intra-team knowledge hiding on creativity at values of learning goal orientation.

Learning goal orientation	Effect	BootSE	BootLLCI	BootULCI	Sig.
−1 SD (−0.861)	−0.218	0.077	−0.359	−0.055	**
Mean (0.000)	−0.168	0.073	−0.305	−0.013	*
+1 SD (+0.861)	−0.119	0.076	−0.260	0.043	n.s.

*N* = 275. Bootstrap sample size = 10,000; BootLLCI = bootstrap lower limit of the 95% confidence interval; BootULCI = bootstrap upper limit of the 95% confidence interval.

Conditional total effects were computed as c’ + (a1 + a2 × W) × b, where c’ = −0.328 is the direct effect of ITKH on creativity, a1 = 0.374 is the effect of ITKH on GenAIKS, a2 = 0.135 is the ITKH × LG interaction coefficient, b = 0.427 is the effect of GenAIKS on creativity, and W denotes the mean-centered value of learning goal orientation. Performance-prove and performance-avoid goal orientations were held at their means.

*p < 0.05, **p < 0.01, n.s., not significant.

**Figure 1:**
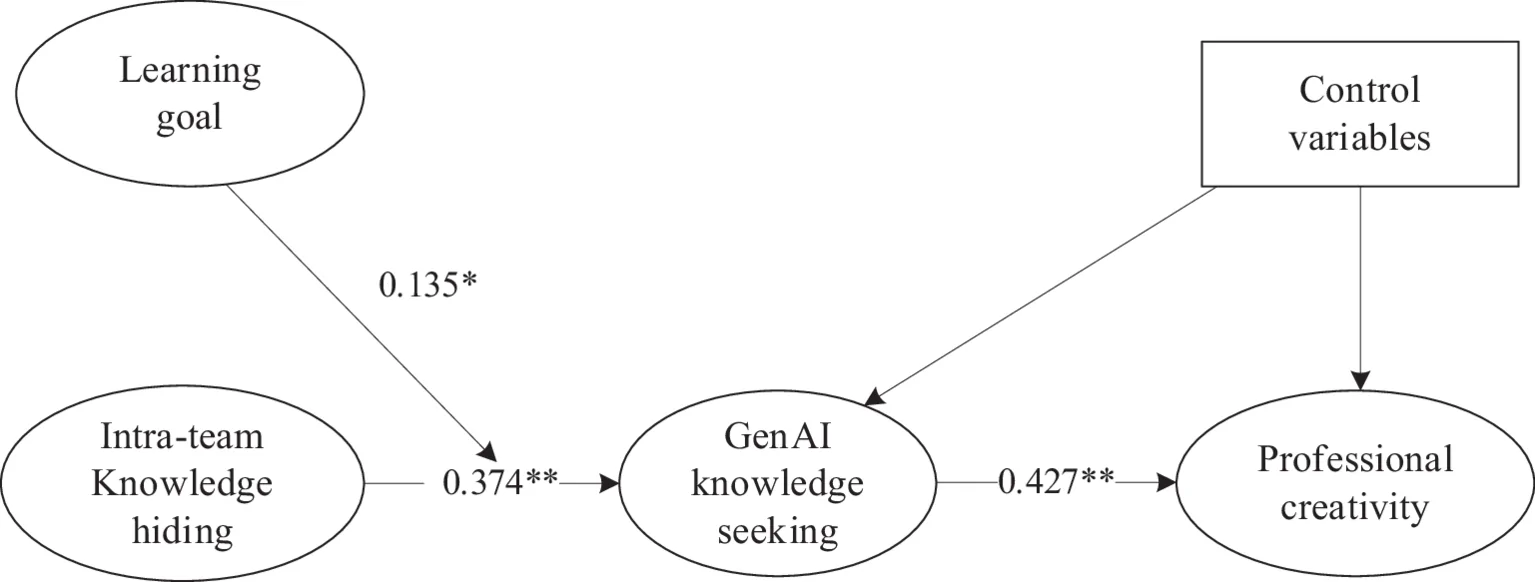
Path modeling results. Only significant paths of interest were reported; full model results can be found in Table 4; control variables were gender, age, education, team tenure, team size, project phase, and three industry dummies; * *p* < 0.05, **p < 0.01, *N* = 275.

We have proposed a positive relationship between intra-team knowledge hiding and GenAI knowledge seeking (Hypothesis 1). According to the results of Model 2, knowledge hiding was positively associated with GenAI knowledge seeking (*b* = 0.374, *p* < 0.01), supporting Hypothesis 1. The positive relationship between intra-team knowledge hiding and GenAI knowledge seeking is consistent with the attention-based explanation developed in this study. When knowledge hiding disrupts access to an expected internal knowledge source, knowledge seekers may redirect their attention toward alternative channels that can help address the resulting informational gap. GenAI provides an immediately accessible and interactive means of acquiring and synthesizing task-relevant knowledge, thereby serving as a compensatory channel when internal knowledge flows are disrupted.

According to Hypothesis 2, GenAI knowledge seeking was positively associated with creativity (*b* = 0.427, *p* < 0.01), and the indirect relationship between intra-team knowledge hiding and creativity through GenAI knowledge seeking was positive and significant (*b* = 0.160, *p* < 0.01). Therefore, Hypothesis 2 was supported. Scholars have empirically emphasized the important roles of external knowledge in fostering individual creativity (Badir et al., 2020; Santangelo and Phene, 2022) and enabling teams to better use internal knowledge to realize creativity (Venkataramani and Tang, 2024). In addition to the enhancing and supplementing roles in facilitating creativity, external knowledge obtained from AI-enabled tools can also benefit employee creativity by filling the knowledge gap resulting from intra-team knowledge hiding.

Regarding the moderating effects, it was found that learning goal orientation was associated with a stronger positive relationship between intra-team knowledge hiding and GenAI knowledge seeking (*b* = 0.135, *p* < 0.05), as well as a stronger positive indirect relationship (*b* = 0.058, *p* < 0.05), supporting Hypotheses 3a and 4a. The practical significance of this interaction is further reflected in the conditional indirect effects: the indirect effect of intra-team knowledge hiding on creativity via GenAI knowledge seeking increased from *b* = 0.110 at low levels of learning goal orientation to *b* = 0.209 at high levels. This finding suggests that learning goal orientation enhances employees’ tendency to engage in GenAI knowledge seeking as a compensatory response to intra-team knowledge hiding. Drawing on the AGT, learning goal orientation emphasizes the acquisition of knowledge and the improvement of skills and abilities (Payne et al., 2007; Hirst et al., 2011). Learning-oriented individuals who experience knowledge hiding are more likely to perceive and appreciate the opportunities provided by external sourcing approaches and engage more GenAI knowledge seeking behaviors, which enables them to overcome the creativity barriers. The moderating effect is plotted in Figure 2.

**Figure 2:**
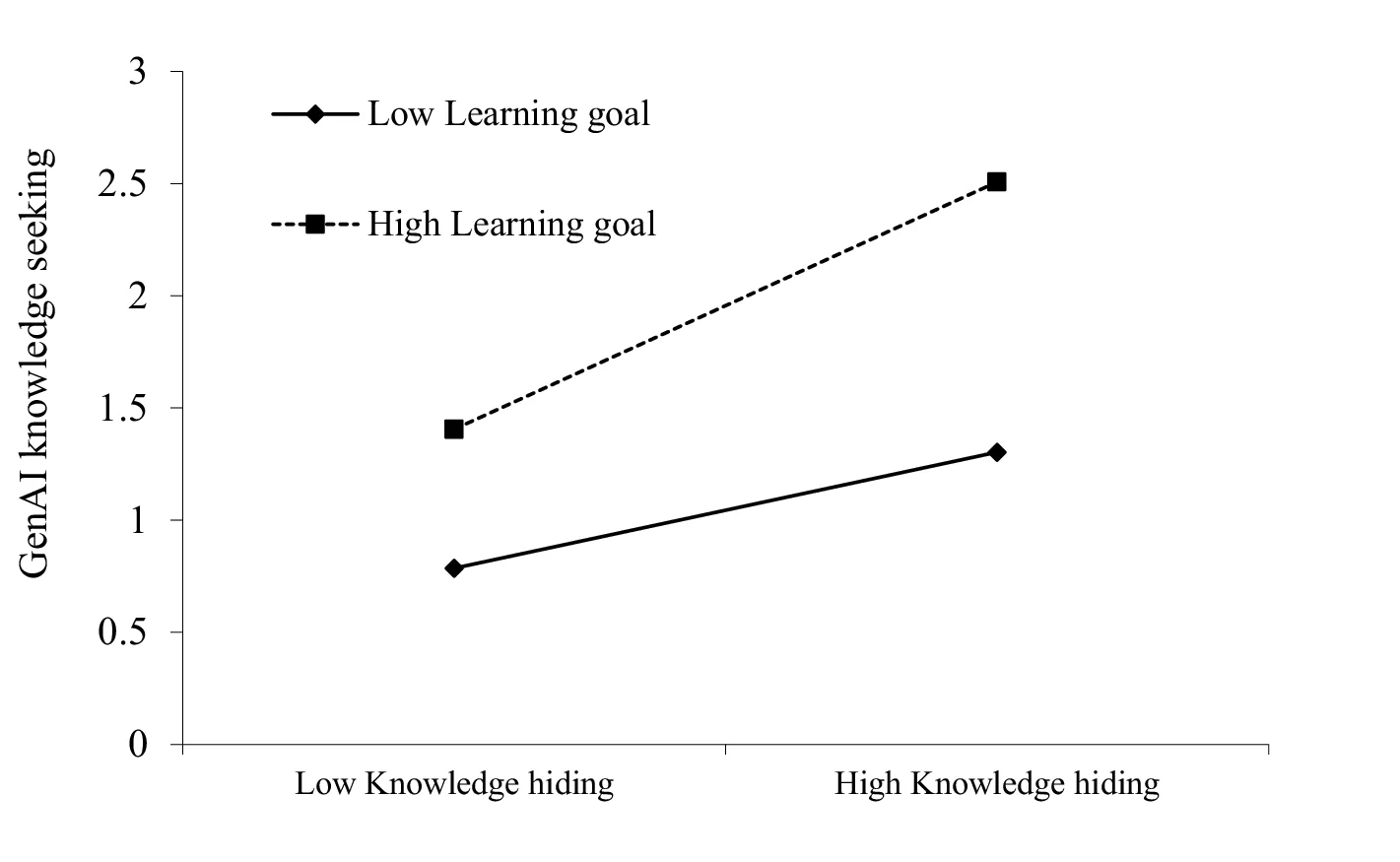
The interaction of intra-team knowledge hiding and learning goal on GenAI knowledge seeking.

Unexpectedly, the moderating effects of performance-prove (*b* = 0.044, non-significant) and performance-avoid goals (*b* = −0.076, non-significant) on the relationship between intra-team knowledge hiding and GenAI knowledge seeking were not significant, rejecting Hypotheses 3b and 3c. Meanwhile, the moderating effects of prove (*b* = 0.019, non-significant) and avoid goals (*b* = −0.033, non-significant) on the indirect relationship between intra-team knowledge hiding, GenAI knowledge seeking, and creativity were not significant either. Therefore, Hypotheses 4b and 4c were not supported. The double-edged sword mechanism of GenAI knowledge seeking may play a role here. On the one hand, GenAI-enabled external knowledge seeking enables knowledge seekers to seek diverse perspectives, experiences, and expertise at their convenience from anywhere (Singh et al., 2018). On the other hand, GenAI knowledge seeking cannot guarantee that every seeker will obtain the desired knowledge even when they have exerted a high level of effort in searching and acquiring knowledge (Wasko and Faraj, 2005). This is due to the fact that participation in online resources is usually an uncertain situation for seekers (Tsai and Kang, 2019), especially for NPD professionals who frequently seek and use complex knowledge. For instance, seekers sometimes lack clear cues regarding the accuracy and contextual relevance of AI-generated knowledge. As a result, prove-oriented individuals can hardly have a stable evaluation on the effectiveness of GenAI knowledge seeking. Moreover, knowledge risks associated with intra-team knowledge hiding motivate avoid-oriented individuals to seek knowledge via GenAI tools; however, the uncertainty and potential risks embedded in AI-generated knowledge may place these employees in a dilemma.

Although Hypothesis 2 was supported, additional analyses revealed that this positive indirect effect coexists with a significant negative direct effect (*b* = −0.328, *p* < 0.01) and a significant negative total effect (Model 1: *b* = −0.182, *p* < 0.05), estimated in a separate model without the mediator. Because this unconditional total effect and the moderated mediation model are not directly comparable, to properly benchmark the total effect against the indirect effect we instead use the conditional total effect reported in Table 5, obtained within the same model; at the mean level of learning goal orientation, this yields a total effect of *b* = −0.168. This pattern, in which the direct and indirect paths carry opposite signs, corresponds to competitive mediation (Zhao et al., 2010), statistically equivalent to a suppression effect (MacKinnon et al., 2000). Because the direct harm (−0.328) exceeds the total effect (−0.168) in magnitude, relying on the total effect alone would understate the damage knowledge hiding does to creativity; the positive indirect effect indicates that GenAI knowledge seeking functions as a genuine but partial compensatory channel rather than reversing this harm. This compensation is also conditional on individual differences: as shown in Table 5, the total effect remains significantly negative for employees low in learning goal orientation [−0.218, 95% CI (−0.359, −0.055)] and at the sample mean [−0.168, 95% CI (−0.305, −0.013)], but is offset to non-significance for employees high in learning goal orientation [−0.119, 95% CI (−0.260, 0.043)].

## 5 Discussion and conclusion

### 5.1 Theoretical implications

The current study can contribute to knowledge management literature in the following ways. First, this study advances creativity research by broadening the focus beyond preventing knowledge hiding to include how individuals respond to and partially compensate for its creativity-related risks in digitally mediated NPD contexts. While previous studies have primarily emphasized the detrimental consequences of knowledge hiding within teams (e.g., reduced knowledge sharing and creativity) (Men et al., 2020; Oubrich et al., 2021; Donate et al., 2022), knowledge hiding is often an inevitable byproduct of task interdependence, time pressure, and resource constraints in project-based NPD work (Zhang and Min, 2024). Addressing this gap, our findings indicate that intra-team knowledge hiding is positively associated with individuals’ engagement in GenAI knowledge seeking, consistent with an attentional shift toward alternative, technology-enabled knowledge sources. This suggests that, beyond seeking to eliminate knowledge hiding, it is also important to understand how individuals respond when access to internal knowledge is constrained.

Second, this study contributes to the literature by uncovering a process-based mediating mechanism through which intra-team knowledge hiding influences individual creativity. Prior research has largely treated knowledge hiding as a direct impediment to creativity by framing it as a breakdown of internal knowledge exchange (Serenko and Bontis, 2016; Connelly and Zweig, 2015). Drawing on ABV, this study demonstrates that intra-team knowledge hiding may redirect individuals’ attention away from internal knowledge sources and toward GenAI-enabled external knowledge channels, thereby identifying GenAI knowledge seeking as a compensatory mediating pathway. This finding extends prior research on external knowledge seeking, which has primarily focused on deliberate efforts to access customers, experts, and other human or organizational sources (Badir et al., 2020; Santangelo and Phene, 2022; Venkataramani and Tang, 2024), by showing that GenAI can function as a technology-mediated external knowledge channel when internal knowledge access is disrupted. Importantly, this mechanism operates as a competitive mediator rather than a reversing force: it mitigates, but does not eliminate, the net creativity cost of knowledge hiding. This refines the prevailing view of knowledge hiding as a uniform impediment to creativity by showing that its influence operates through two countervailing pathways simultaneously, with the compensatory pathway partially offsetting, though not reversing, the direct harm.

Third, this study contributes to the knowledge management literature by identifying learning goal orientation as an important boundary condition that explains when intra-team knowledge hiding is more likely to trigger GenAI knowledge seeking. While prior research has acknowledged that individuals differ in their reactions to knowledge hiding (Lin et al., 2023; Connelly and Zweig, 2015), little is known about how motivational orientations influence technology-enabled coping responses in the face of knowledge hiding. Drawing on achievement goal theory (AGT), our findings reveal that learning goal orientation strengthens the positive relationship between intra-team knowledge hiding and GenAI knowledge seeking. This finding extends prior research on individual responses to knowledge hiding, which has documented various reactions such as reciprocal hiding and reduced support (Connelly and Zweig, 2015; Lin et al., 2023), by identifying motivational orientation as a factor explaining why individuals differ in how they respond. It also complements emerging research on GenAI use by demonstrating that engagement with GenAI as an alternative knowledge source depends on individual motivational differences rather than arising uniformly across employees.

### 5.2 Practical implications

Our study also provides several managerial implications to micro innovation management, especially for individual creativity in NPD project teams. First, GenAI knowledge seeking can provide a compensatory pathway through which professionals partially alleviate the creativity-related constraints associated with intra-team knowledge hiding by accessing external, technology-enabled knowledge sources. When internal knowledge sourcing is disrupted, GenAI tools can help redirect individuals’ attention toward alternative knowledge channels that support creative task completion. To facilitate timely and effective use of GenAI knowledge seeking, organizations may provide targeted training programs and share successful application cases to familiarize professionals with GenAI tools and enhance their confidence in using them to address knowledge-related challenges in creative work (Zhang et al., 2023).

Second, our findings suggest that NPD professionals with a strong learning goal orientation are more capable of mitigating the creativity risks resulting from knowledge hiding by actively engaging in GenAI knowledge seeking. Therefore, organizations are encouraged to create a work environment that supports learning-oriented behaviors and reinforces employees’ motivation for continuous learning. Prior research suggests that learning goal orientation can be shaped by factors such as openness to innovation, developmental performance feedback, and beliefs in one’s own capabilities (Chen and Liu, 2018; Annosi et al., 2020). Accordingly, organizations that rely on NPD projects for innovation may implement developmental feedback, learning-supportive practices, and opportunities for capability development to encourage adaptive responses to knowledge constraints.

Third, internal and external knowledge sourcing can be viewed as two interrelated and complementary approaches to supporting creativity (Wang et al., 2014; Badir et al., 2020). Relying exclusively on either internal knowledge exchange or external knowledge sourcing is unlikely to maximize creative outcomes. While GenAI knowledge seeking plays an important role in compensating for knowledge loss caused by intra-team knowledge hiding, project managers should guide professionals to balance GenAI-enabled external knowledge seeking with internal knowledge interactions to ensure effective integration of diverse knowledge inputs in NPD tasks. Importantly, investing in GenAI capabilities should not substitute for addressing the conditions that give rise to knowledge hiding. Because the overall relationship between knowledge hiding and creativity remains negative, organizations that rely solely on technology-enabled compensation may mask rather than resolve the underlying problem.

### 5.3 Limitations and future research directions

Like many other studies, this study had several limitations. Firstly, although a multi-wave, multi-informant design was employed, the study remains observational, which limits our ability to establish causal relationships. To obtain a more robust understanding of the causal paths, future studies could consider utilizing a longitudinal survey approach, collecting more data at multiple time points.

Secondly, although this study controlled for several demographic and project characteristics, several theoretically relevant variables were not included. Prior GenAI experience may shape individuals’ familiarity with and ability to evaluate AI-generated knowledge, task complexity may influence both knowledge-seeking behavior and creative performance, and access to alternative external knowledge sources may substitute for or complement GenAI knowledge seeking. The omission of these factors may have influenced the estimated relationships, and future research should incorporate them as controls or boundary conditions.

Thirdly, the empirical data were collected from Chinese NPD teams through the alumni network of a single university in eastern China. The sampled firms may therefore be more innovation-oriented than the average NPD organization, and the sample was predominantly male and highly educated. Cultural differences in motivations for interpersonal knowledge transfer may also shape responses to knowledge hiding (Zhang and Min, 2024). The findings should thus be interpreted as applying primarily to innovation-oriented NPD teams in Chinese technology firms staffed by highly educated professionals, rather than as generalizable to all NPD professionals or organizational settings. Future research should replicate this study using broader sampling frames across regions, industries, and cultural contexts.

Fourthly, this study focused on the creative consequences of project professionals’ knowledge hiding. In addition to creativity, routine task performance is also an integral component of organizational performance (Madjar et al., 2011). Future research should explore the task related effect of knowledge hiding and its coping strategies to provide further empirical support for managing project knowledge hiding.

Fifthly, most studies on knowledge hiding rely on self-report scales to measure the extent of knowledge hiding (Connelly and Zweig, 2015; Connelly et al., 2012). To ensure the validity and applicability of our findings, researchers should reevaluate our results by developing observed knowledge hiding items.

Sixthly, our measure captures knowledge access, that is, the frequency and intensity of engagement, rather than the depth of interaction or the subsequent evaluation, recombination, and exploitation of the knowledge obtained (Nonaka, 1994; Zahra and George, 2002). Future research could develop finer-grained measures and model these downstream stages directly.

Finally, this study primarily examined general knowledge and did not differentiate between explicit and tacit knowledge. A more comprehensive contribution can be made by considering the various types of hidden knowledge.

## Data Availability

The raw data supporting the conclusions of this article will be made available by the authors, without undue reservation.
