# Supplementary material for: Mitigating creativity risks of knowledge hiding through GenAI knowledge seeking: moderating roles of goal orientation
Source: Front Psychol. 2026 Sep 22;17:1874454. doi: 10.3389/fpsyg.2026.1874454 (PMC13640130; doi:10.3389/fpsyg.2026.1874454)
Supplement: Supplementary file 1 [file Table_1.docx]

**Appendix A. Cluster-robust re-estimation**

Although all focal constructs were specified and measured at the individual level, respondents were nested within project teams (275 employees in 167 teams; 108 two-member and 59 single-member teams; average cluster size = 1.647). We therefore re-estimated the focal model with project-team cluster-robust (sandwich) standard errors, treating project team as the clustering unit. Table A1 reports the resulting estimates.

**Table A1**

**Focal model estimates with cluster-robust standard errors**

| **Path / effect** | ***b*** | **SE** |
| --- | --- | --- |
| **Structural paths** | | |
| KH → GenAI seeking (a) | 0.374** | 0.040 |
| GenAI seeking → Creativity (b) | 0.427** | 0.078 |
| KH → Creativity (c′, direct) | −0.328** | 0.078 |
| KH × LGO → GenAI seeking | 0.135** | 0.046 |
| KH × PPG → GenAI seeking | 0.044 | 0.085 |
| KH × PAG → GenAI seeking | −0.076 | 0.075 |
| **Effect decomposition (KH → Creativity)** | | |
| Indirect (a × b) | 0.160** | 0.034 |
| Direct (c′) | −0.328** | 0.078 |

*Notes:* Unstandardized path estimates (b) and standard errors (SE) from Mplus 8.3 (MLR), re-estimated with cluster-robust standard errors (TYPE = COMPLEX; project team as the clustering unit). All point estimates remain consistent with those in the main text, and every path retains its sign and significance. KH = intra-team knowledge hiding; LGO, PPG, PAG = learning, performance-prove, and performance-avoid goal orientation.

N = 275, * p < .05, ** p < .01.
